# Study of Microchannels Fabricated Using Desktop Fused Deposition Modeling Systems

**DOI:** 10.3390/mi12010014

**Published:** 2020-12-25

**Authors:** Muhammad Asif Ali Rehmani, Swapna A. Jaywant, Khalid Mahmood Arif

**Affiliations:** Department of Mechanical and Electrical Engineering, SF&AT, Massey University, Auckland 0632, New Zealand; m.a.a.rehmani@massey.ac.nz (M.A.A.R.); s.jaywant@massey.ac.nz (S.A.J.)

**Keywords:** additive manufacturing, microfluidics, microchannel, curved microchannel, fused deposition modelling, 3d printing

## Abstract

Microfluidic devices are used to transfer small quantities of liquid through micro-scale channels. Conventionally, these devices are fabricated using techniques such as soft-lithography, paper microfluidics, micromachining, injection moulding, etc. The advancement in modern additive manufacturing methods is making three dimensional printing (3DP) a promising platform for the fabrication of microfluidic devices. Particularly, the availability of low-cost desktop 3D printers can produce inexpensive microfluidic devices in fast turnaround times. In this paper, we explore fused deposition modelling (FDM) to print non-transparent and closed internal micro features of in-plane microchannels (i.e., linear, curved and spiral channel profiles) and varying cross-section microchannels in the build direction (i.e., helical microchannel). The study provides a comparison of the minimum possible diameter size, the maximum possible fluid flow-rate without leakage, and absorption through the straight, curved, spiral and helical microchannels along with the printing accuracy of the FDM process for two low-cost desktop printers. Moreover, we highlight the geometry dependent printing issues of microchannels, pressure developed in the microchannels for complex geometry and establish that the profiles in which flowrate generates 4000 Pa are susceptible to leakages when no pre or post processing in the FDM printed parts is employed.

## 1. Introduction

Microfluidics, the science and technology of manipulating fluids at micro to millilitre scale through internal features, has been applied in various applications such as point-of-care diagnostic tools, therapeutic devices, and air and water quality monitoring methods [1,2,3,4,5,6]. Currently, popular manufacturing techniques for producing microfluidic devices include soft lithography [7], paper microfluidics [8,9], micromachining [10], injection moulding [11], hydrid paper based open channel microfluidics [12], etc. Softlithography using polydimethylsiloxane (PDMS) micro-moulding is widely used method as PDMS is optically transparent, chemically inert, and gas permeable material with low-surface energy [13]. However, most of these methods are expensive, time-consuming, require multi-step processing in a cleanroom. Additionally, it is quite difficult to change device design [14]. Recently, 3-dimensional printing (3DP) is rapidly gaining attention in the field of microfluidics. The prominent fabrication techniques for microfluidics are fused deposition modelling (FDM), selective laser sintering (SLS), stereolithography (SLA), Xurography and inkjet printing [15,16,17,18,19]. These methods have various advantages like automated fabrication, cost-effectiveness, availability of a wide range of materials, single-step procedure, etc. [13,20,21,22]. Moreover, 3DP does not require a photomask, photoresist, or access to a cleanroom. This allows a major reduction in the material cost, creates the possibility of mass manufacturing, and saves significant development time [13].

The literature shows that 3DP methods such as FDM [1,23,24,25,26,27], SLA [28,29], and inkjet printing [30] have been used in the field of microfluidics. Symes et al. [2] printed the reagents directly into a 3D reactionware for organic and inorganic synthesis using FDM. Similarly, Anciaux et al. [31] fabricated a micro free-flow electrophoresis device with a consumer-grade 3D printer. The performance of FDM, SLA, and polyjet printers have been compared with the help of microfluidic chip including sealed and open-channel micro-mixers [26,32,33,34].

The performance of 3D-printing devices depends upon the choice of process. In microfluidic applications, Stereolithography (SL) printing has been employed widely which works on the principle of curing a photopolymer resin layer-by-layer [35,36]. However, conventional SL resin is susceptible to toxicity and is not biocompatible [37]. Development of non-toxic and biocompatible SL resin can be expensive and requires rigorous experimentation. In this regard, FDM printing is cost-effective and offers a choice of the biocompatible materials. Moreover, the low cost of FDM printer makes it one of the promising 3D printing technologies for fabricating non-toxic devices, which are essentially required for Lab-on-a-Chip applications. Despite the aforementioned benefits of FDM additive manufacturing, there are applications where other AM techniques can provide better microfluidic devices to study, investigate or harness the physical phenomenon at micro or sub micro level. In this regard Table 1 summarizes the methods of fabrication, advantages and limitations of prominent AM and other microfluidic fabrication techniques which can be utilized to fabricate different functional microfluidic devices of interest.

**Figure 1:**
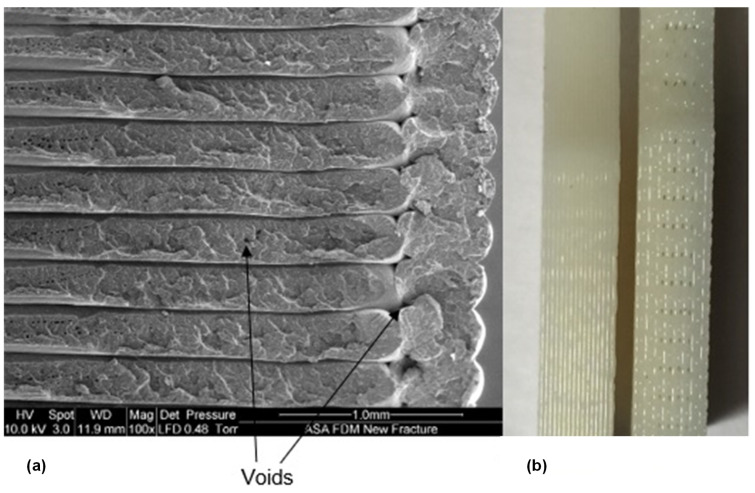
Voids in the FDM printed parts. (**a**) Scanning Electron Microscope (SEM) image showing usual voids inherent to the FDM process. (**b**) Optical image of a dog-bone sample with clearly seen voids.

Most of the microfluidic devices involve micro-mixing of reagents. In these applications, the process is primarily dependent upon diffusion of two different flows. The flow rate and channel length are important parameters to achieve effective mixing [32]. Additionally, the design of microchannel, it’s size, and the effect of fluid pressure should be considered to explore the possibilities of leakage through the microfluidic devices. Furthermore, FDM printers leave voids while printing the part (Figure 1) as layers are not uniformly bound. Hence, it is also important to characterise the microchannels for their mechanical characteristics.

In this paper, we explore the possibilities of using FDM technology for developing the internal features of the microfluidic devices with non-transparent materials. The internal features consisted of in-plane profiles (i.e., linear, curved and spiral microchannels) as well as microchannels with varying cross-section in the build direction (i.e., helical microchannel).

The comparison of pressure developed was performed based on the minimum possible channel size, fluid flowrate, and leakage in the microchannel body. Furthermore, FDM printed parts have been analysed to observe the absorbance of the fluid due to the presence of voids in the layers. Also, the effect on the microchannels part weight with respect to flowrate after cleaning it with acetone has been included and discussed.

## 2. Materials and Methods

Two FDM printers with 0.4 mm nozzle were used in this study: (1) a Tiertime UP 02 (referred as UP02) and (2) an Original Prusa i3 MK3S (referred as Prusa). The UP02 was controlled through UPStudio software and the Prusa was used with Prusa Slicer and Pronterface softwares. The models for 3D parts were constructed using parametric 3D modelling software Autodesk Inventor Professional 2020. One of the main requirements for the microfluidic application in biomedical applications is their biocompatibility [49,50,51]. For this purpose, we chose polylactic acid (PLA) printing material, which is biodegradable and does not leave a hazardous footprint in the environment. Moreover, it has low warping deformation and overall printed parts exhibit excellent dimensional accuracy and quality [52,53]. The spool of PLA filament having a diameter of 1.70 mm was used to deposit the layers of modelled microfluidic channels. Pico Plus Syringe Pump from HARVARD APPARATUS was used for injecting the water at different flow rates. Pressure measurements were performed with the help of a PX3 Series heavy-duty Honeywell pressure transducer having pressure measurement range of 0 to 8600 kPa with total error band (TEB) of ±1% full-scale span (FSS). Sartorius ENTRIS64-1S analytical balance was used for the weight analysis.

### 2.1. Fabrication of Microchannels

Four types of microchannels (linear, curved, spiral along layer and helical, where microchannel is also moving perpendicular to the layer) were designed with different diameter sizes. These microfluidic channels were fabricated using FDM. Different flow rates were applied on the channels for the pressure measurement. The FDM printer was optimised with nozzle temperature at 207 ${}^{{}^{\circ}}$C and platform temperature at 68 ${}^{{}^{\circ}}$C to achieve the best results during the printing process with both the FDM printers. Other process parameters and specifications of the printers are summarized in Table 2.

**Figure 2:**
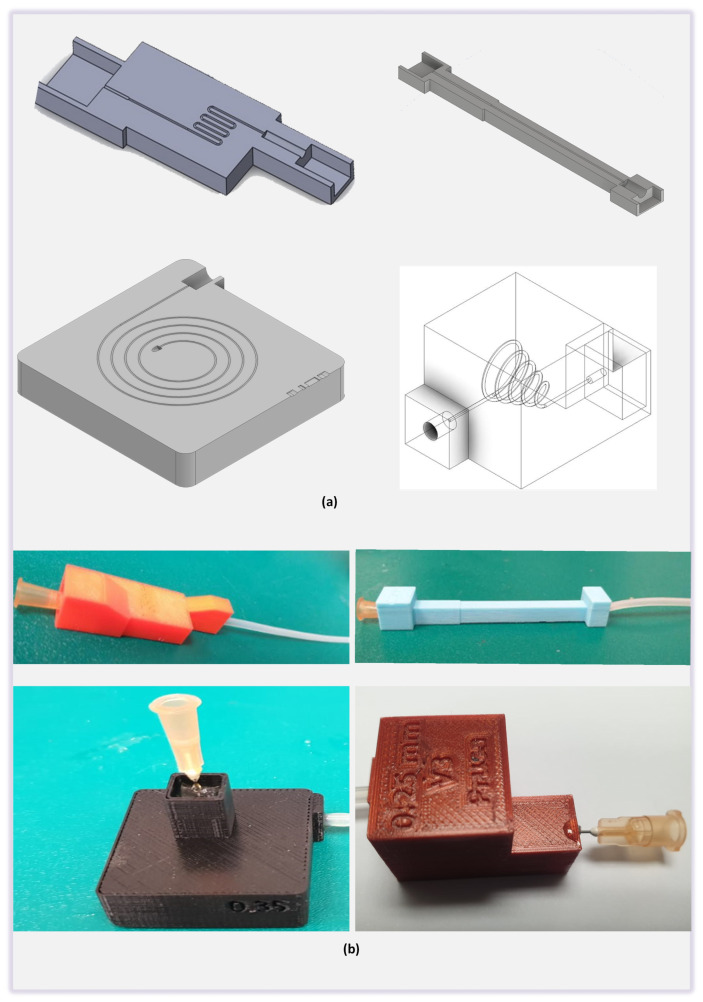
(**a**) CAD models of the linear, curved, spiral and helical microchannels (**b**) Pictorial view of the connected inlet and outlet ports of the FDM printed linear, curved, spiral and helical microchannel.

The channel design consisted of an inlet port, the main channel, and the outlet port. The inlet port is connected with the syringe pump which is then connected with the pressure reservoir. The top of the pressure reservoir is connected with the pressure sensor. Outlet port of the pressure sensor reservoir was connected with a luer lock connector which can be easily attached via a conventional hypodermic needle bonded with the inlet port of the fabricated microchannels. Any change in the pressure induced by the change of the flowrate at a specific microchannel diameter was then recorded by the pressure sensor. The output signal of the pressure sensor in voltages was then converted to the pressure through the characteristic curve of the sensor provided by the OEM of the sensor. The inner diameter of the inlet port of the printed microchannel was kept constant at 0.55 mm so that the 25-gauge hypodermic needle can easily be inserted to the inlet port of the microchannel. whereas, the diameter of the main channel was fabricated with various diameter sizes (0.25 mm, 0.3 mm, 0.35 mm, 0.4 mm, 0.45 mm, and 0.5 mm). The connection with the inlet port of the printed microchannel was secured by using the 3M™ Scotch Weld™ epoxy adhesive DP125 having a tensile strength of 17.23 MPa with the hypodermic needle. The outlet port with a diameter of 0.3 mm was used to connect the silicone tubing. In the linear channel (Figure 2a,b), length of the main channel was kept at 50 mm and the distance between each of the 4 walls was set at 5 mm to avoid immediate leakage possible depending upon the control on the print settings from the slicer software. On the other hand, for the curved channel, the path consisting of 6 half circle turns of 1 mm diameter and 7 straight paths with the total linear length of 50 mm. Spiral channel also designed with the same philosophy of keeping the overall length of the microchannel up to 50 mm while the injection port was matched with the syringe outer diameter.

### 2.2. Characterisation

The printed results from the FDM printer shows that the accuracy of the printer not only varies with the type of the printer but it also varies if the microchannels are along the print layers or perpendicular to the print layers. For instance, when the microchannels are along the printing layers the smallest achievable microchannels with the off-shelf Up printer was around 250 μm whereas when the geometry of the channel changes and microchannels follows the path which is perpendicular to the build layers the size of the microchannels needs to be increased to 900 μm to be able to achieve the flow across the microchannels while keeping the print settings similar to the other geometry. This study provides a practical demonstration of the same through pressure developed across the flow of the minimum possible channel size.

To ascertain the quality of the print, we measured the surface roughness of the printed parts. The measurement of the printed specimen was done by a surface roughness profilometer from STARR instrument model RTD-210. The surface roughness profilometer stylus measurement probe had a low contact force of 4 mN and a tip radius of 5 μm having 90${}^{{}^{\circ}}$ cone angle of diamond tip. The profiling speed for the test was maintained at 0.5 mm/s resulted in the overall accuracy of 4 nm/±40 μm in Z-axis and Gaussian filter has been selected to suppress the noise during the measurement of the surface roughness. All the printed FDM parts were printed at an angle of 45${}^{{}^{\circ}}$ therefore, the tip of the profilometer was held at an angle of 45${}^{{}^{\circ}}$ for each measurement with respect to the printed surface. The tracing path was auto-levelled with a sampling cut-off length of 0.8 mm and measuring range of 4 mm.

**Figure 3:**
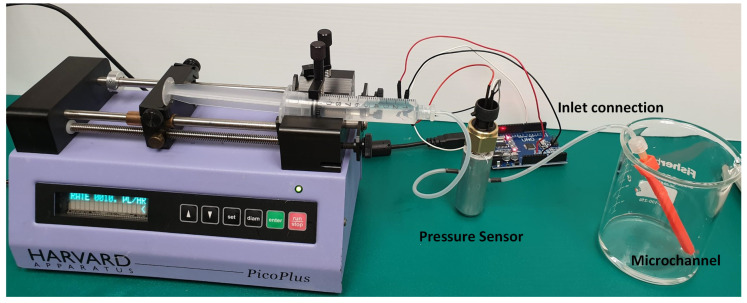
Pressure measurement experimental setup.

Linear, curved, and spiral microchannels have been characterised in the paper. The characterisation was performed to obtain the minimum possible diameter with each technology. Additionally, the maximum possible flow rate without any leakage through the channels was studied. The experimental set up is explained in Figure 3. Initially, all the printed channels were cleaned with compressed air jets. To ascertain the leakage from the body of the microchannel the consumed water was dyed blue and was injected at different flow rates (varying from 0 to 40 μL/min in steps of 5) was injected at the input port with the help of the syringe pump. A disposable, 60 mL syringe was actuated on the syringe pump. For each measurement, the syringe pump was operated to dispense enough liquid so that a stable reading at the pressure sensor can be recorded. A pressure sensor was placed in-between the syringe pump and the input port of the channel for corresponding pressure measurement. The pressure was obtained as voltage value which, in turn, was converted into pressure value (Pa) using the data-sheet. The change in flow-rate at the input port resulted in a change in pressure at the input port and leakage was observed in the microchannel.

Generally, the voids present in the FDM printed parts are responsible for leakage or water retention. Hence, the effect of acetone cleaning on the microchannel surface was analysed. This was based on the idea that the additives in PLA may react with acetone and melt to fill the gaps and voids inside the microchannel. Acetone was circulated through the FDM printed microchannels for 10 min. Furthermore, printing accuracy test for FDM printer was performed by simultaneously printing 8 microchannels of each size and weighing each part separately on the analytical balance.

## 3. Results and Discussion

**Figure 4:**
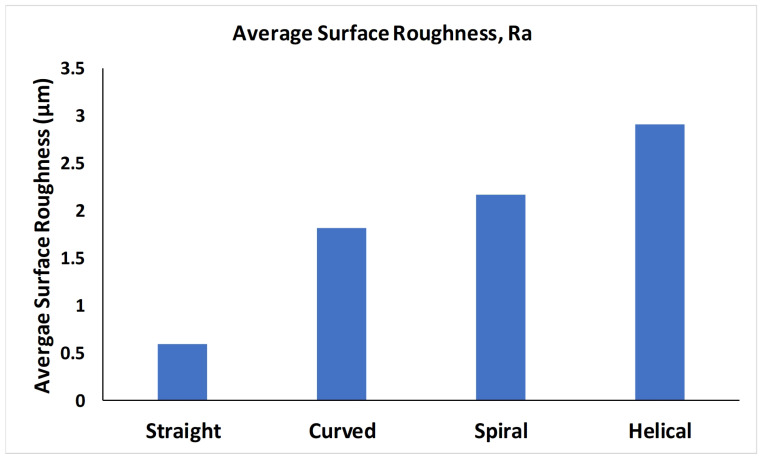
Average surface roughness (Ra) of straight, curved, spiral and helical microchannels.

The average surface roughness of each type of microchannel fabricated with FDM printer has been provided in Figure 4 and the respective parameters of surface roughness profile are mentioned in Table 3. The peak height for all the channels was within ±10 μm. Surface roughness of each print having the same microchannel design is averaged to calculate the resultant surface roughness of the parts. The variation of the surface roughness is due to the change in the profile of the each specimen and printing orientation of the specimen or profile feature. Surface roughness values depends on the print setting such as the nozzle diameter, layer thickness, infill properties and layer over-lapping.

**Figure 5:**
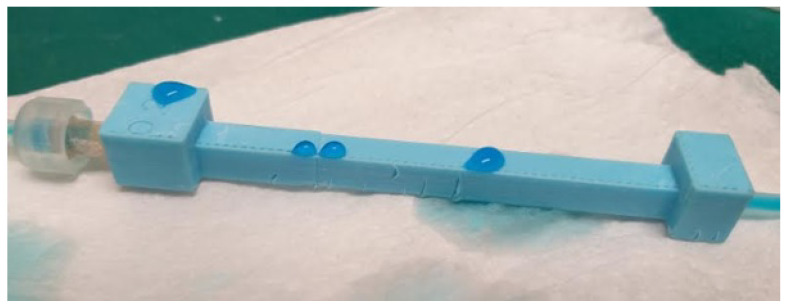
Leakage in the FDM printed linear microchannel.

The microchannels created using the FDM method having diameters ranging from 0.25 mm to 0.5 mm did not have any blockages. The water was passed through the FDM method basd microchannels to study the effect of various flow rates on the leakage. Figure 5 shows the leakage across the body of the FDM printed linear microchannel when the pressure developed along the microchannel is excessive at a particular flow rate and instigates the leakage from the layers of the microchannel body.

The accuracy of the printer to replicate the channel was also determined in the paper. This was achieved by printing a total of 8 samples of each diameter (0.25, 0.3, 0.35, 0.4, 0.45, and 0.5 mm) at the same time individually. A default base support structure was carefully removed using scrapper and filament cutter to ensure to obtain the accurate readings without damaging the part. Each microchannel weight was individually recorded on the analytical scale. This test recorded a total of 144 channels being printed for three different micro-channel designs (linear, curved, spiral along layer) and 40 prints for the helical design where microchannel also moves perpendicular to the layers. At the end of printing, we had 8 replicates of each diameter for each design. However the size of the microchannel for the microchannel geometry which changes perpendicular to the layer has different channel size. Since, the printed results from the FDM printer shows that the accuracy of the printer not only varies with the type of the printer but it also varies if the microchannels are along the print layers or perpendicular to the print layers. For instance in case when the microchannels are along the printing layers the smallest achievable microchannels with the off-shelf Up printer was around 250 μm where as the geometry of the channel changes and microchannels follows the path which is perpendicular to the build layers the size of the microchannels needs to be increased to 500 μm to be able to achieve the flow across the microchannels while keeping the print settings similar to the other geometry. This study provides the practical demonstration of the same through pressure developed across the flow of the minimum possible channel size.

**Figure 6:**
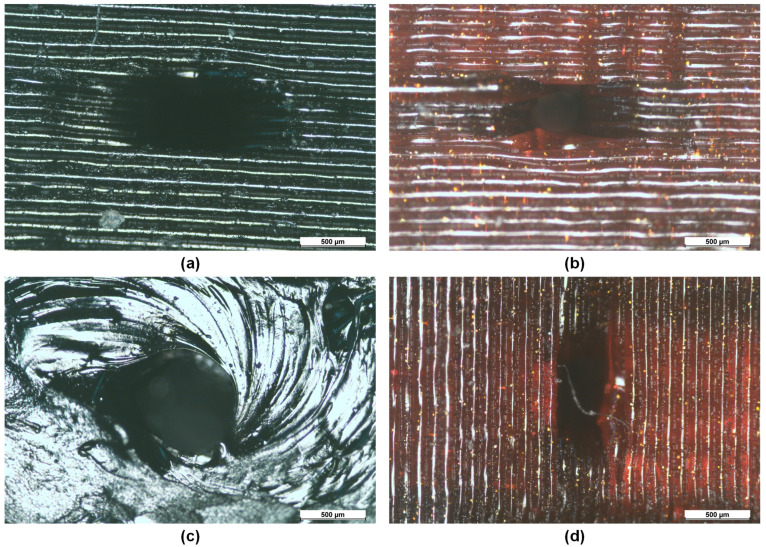
Comparison between printing with Prusa and UP02: (**a**) Horizontal printing by UP02 (**b**) Horizontal printing by Prusa (**c**) Vertical printing by UP02, and (**d**) Vertical printing by Prusa.

Moreover, the accuracy of the print also varies while printing with the Tiertime UP printer and Prusa printer. We have chosen the marlin firmware to generate the G-code which is generally the default selection for the prusa slicer. Whereas, the Tiertime UP printer has the proprietary slicing to generate the layer instructions for the printer. It can be evident from Figure 6 that Tiertime UP printer showed better printing resolution for printing the inlet port of the injection (0.5 mm diameter). The slicing and subsequent printing from the Prusa printer resulted in the smaller inlet port. However, the Prusa slicer allows changes for the setting of prints and is considered to be one of the best open-source slicing software available. From the review of the sliced features, it is evident that the layers show the formation of the inlet ports but the same is not translated in prints by the Prusa i3 MK3S printer. We were unable to test the same slicing on the Tiertime UP printer as the printer does not work with Prusa slicer or any other open source slicing software. It can be inferred from the printing results that the accuracy of the Tiertime UP printer in the category of the low budget 3D printers the accuracy is better when features are printed along the printed layers, however, the slicing software does not allow myriads of parameters which can be adjusted from the open-source Prusa slicer. For our application of microchannels, the accuracy is more important and therefore, the results from the printed microchannels from Tiertime UP printer are more accurate than the Prusa printer.

**Figure 7:**
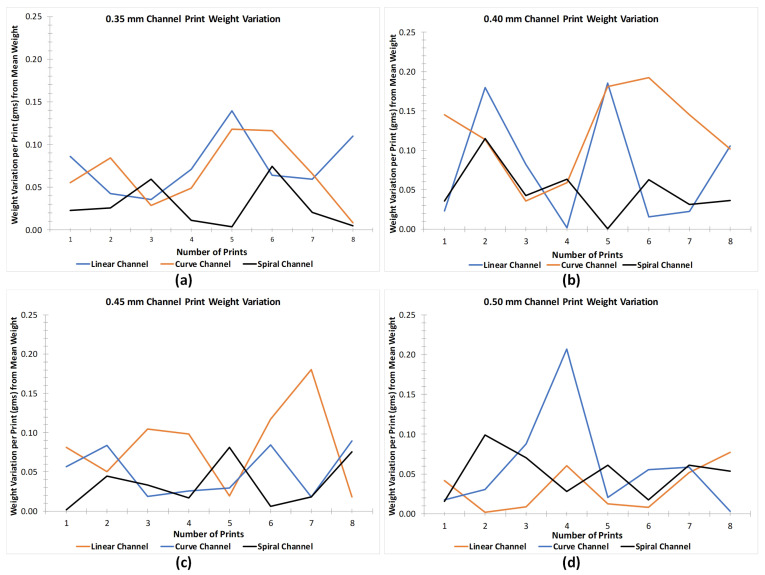
Weight variation of linear, curved and spiral microchannels with diameter of (**a**) 0.35 mm, (**b**) 0.4 mm, (**c**) 0.45 mm, and (**d**) 0.5 mm for 8 consecutive prints.

Once the printing of microchannel parts was completed, the base support structures were removed by using mechanical tools (scrapper and filament cutter). Each microchannel weight was individually recorded on Sartorius ENTRIS64-1S before applying adhesives and attachments to connect the fluidic ports. It was ensured that no channel was damaged while removing the support. This test recorded a total of 48 accurate prints of one type of microchannels. The reason to print 8 microchannels of same diameter, per each type of microchannel, was to evaluate the effect of adsorption or leakage of fluid once the flow rate was increased. As our experiments were composed of evaluating the pressure developed in the microchannels from 5 μL/min to 40 μL/min (with a step of 5 μL/min), 8 prints of each type of microchannel were necessary to be fabricated. Figure 7 illustrates respective graphs of linear, curved and spiral microchannels with different diameters (0.25 mm, 0.3 mm, 0.35 mm, 0.4 mm, 0.45 mm and 0.5 mm). The weight variation of 0.25 mm and 0.30 mm microchannels were removed as the leakage from these microchannels was excessive even at the low flow rates. The actual weights of all the channels were plotted along the average line to represent the deviation in the weights. It is evident from the Figure 7 that variation in weight is negligible among the microchannels. It was also observed that with proper scrapping and removal of support structures the printer could give an excellent precision (with variation of just 0.6%) in both microchannel designs.

**Figure 8:**
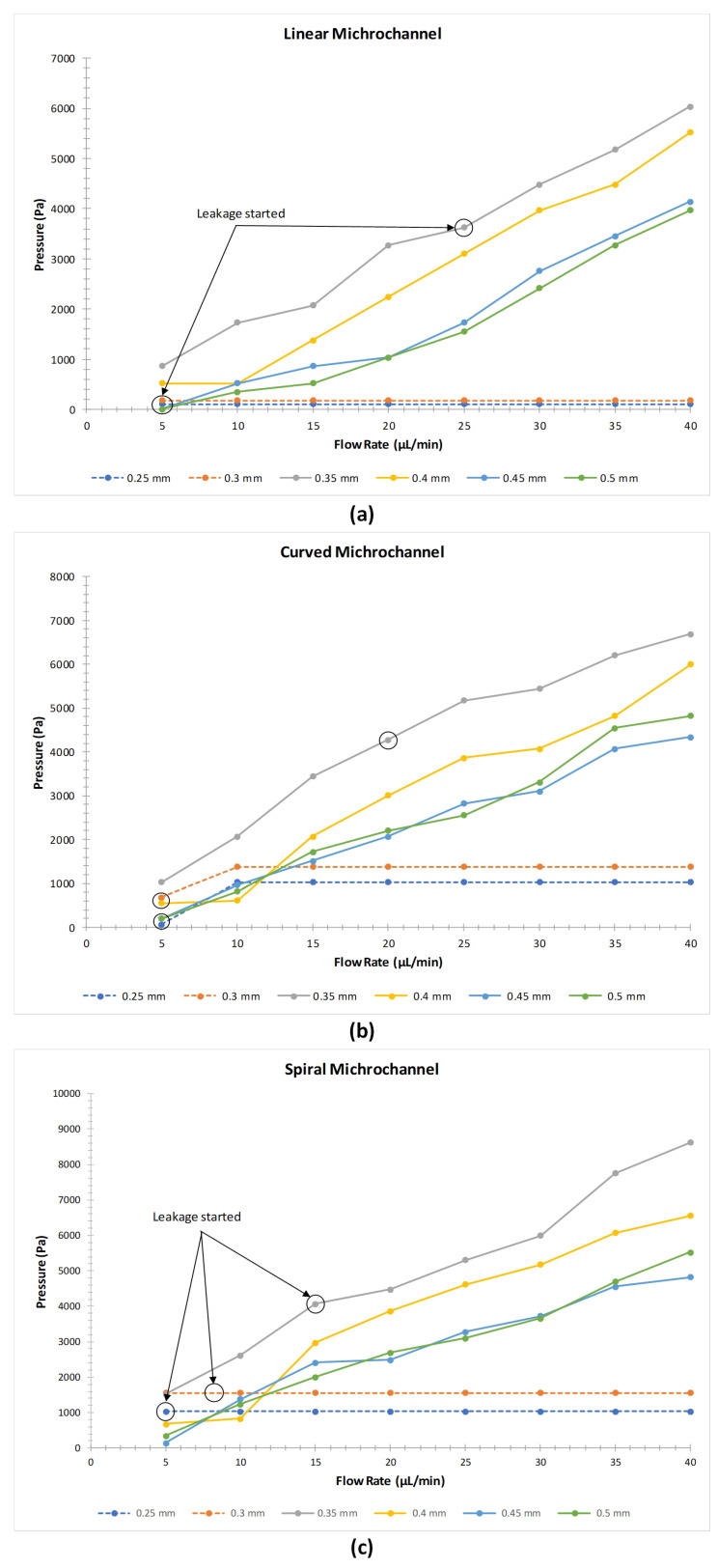
Change in flow rate vs developed pressure at the inlet port of (**a**) linear microchannel, (**b**) curved microchannel and (**c**) spiral microchannel.

The graphs (Figure 8a–c) explain the relationships between the flow rate and the pressure developed in the microchannel with respect to the diameter of the linear, curved, and spiral microchannel respectively. As depicted in the graphs there is a proportional relationship between the flow rate and the pressure. As the flow rate is increased the pressure developed in the channel also increases however, due to the channel length of each FDM printed microchannel and the channel routing the pressure developed is different for each type of microchannel.

It has been observed that all the microchannels with a diameter of 0.3 mm and less started to leak even at minimum flow rates. There is no leakage observed in linear and curved microchannels with a diameter of 0.4, 0.45 and 0.5 mm up to the flow rate of 40 μL/min. This shows that the microchannel having a diameter greater than 0.4 mm can be used for various microfluidic drug delivery applications under a controlled flowrate. However, in the linear and curved microchannel with a diameter of 0.35 mm, no leakage has been observed until the developed pressure reached to 4000 Pa. Above this pressure, the body of the microchannels in both the designs started to leak from the top and bottom side near the inlet port for a diameter of 0.35 mm microchannel. In the case of spiral microchannels, leakage has been observed in 0.35 mm channel diameter but the inception of the leakage started at a lower flow rate when compared with the linear and curved channel. Moreover, it is observed that more pressure is developed across the channels of the curved and spiral microchannels when compared to linear microchannel at the same flow rate. This is due to the constriction in the microchannel due to the geometrical changes in each of the printed layer as the shape of the microchannel was varying along the printed lines. It is therefore, established that the pressure above 4000 Pa in the fluidic channel can result in the leakage from the FDM printed parts for straight, curved and spiral microchannels for microchannels sizes less than 0.4 mm. Table 4 provides the values of pressure developed against the applied flow rate in the microchannels where the cells highlighted in red indicate the flow rates at which the microchannels started to leak.

**Figure 9:**
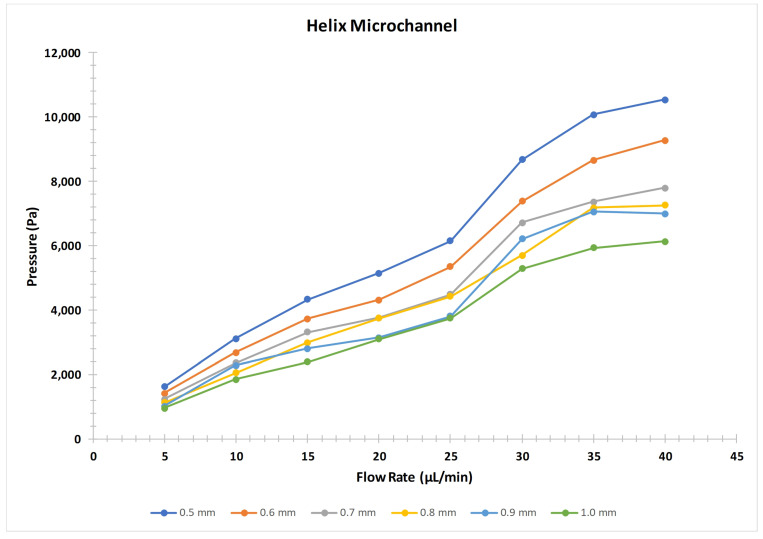
Helical microchannel pressure profile with respect to flow rate.

As discussed earlier the helical microchannel was unable to be printed without the constriction due to the complex profile, therefore the pressure profile of the helical microchannel was not discussed along with the other profiles. We were able to get the flow from the helical profile for microchannel diameter of 500 μm or above. Six profiles with the increment of 100 μm were printed till 1 mm microchannel for the helical profile. The pressure developed in the helical profile was much greater than the other profiles; though, the size of the microchannel was also greater than the other profiles. It was observed that the helical profile resulted in about an average of 2.45 times the pressure generated by the similar size microchannel for spiral profile when compared for the 500 μm size microchannel. This shows that the mapping of the irregular or complex geometrical shapes cannot be exactly replicated during the printing process by FDM printers (Figure 9).

The mapping of the geometrical profile can also be confirmed by the optical microscopic images of the two microchannel holes when the orientation of the printed part is changed during the printing process. Figure 6 shows the optical image of the 500 μm size microchannel when oriented horizontally and vertically during the FDM printing process. The optical image of the printed 500 μm size microchannel corroborates that the internal feature cannot be mapped exactly as designed in the CAD software and due to the same the helical profile offers more constriction in the flow of liquid when compared with the other profiles.

**Figure 10:**
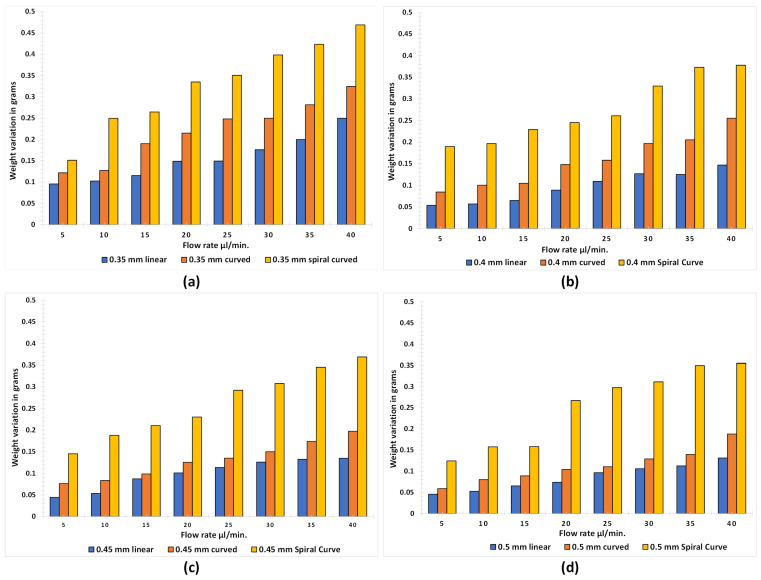
Weight analysis of linear, curved, and spiral microchannels with diameter size of (**a**) 0.35 mm, (**b**) 0.4 mm, (**c**) 0.45 mm, and (**d**) 0.5 mm.

Since, the FDM parts contains voids in between the printed layers therefore, it is natural for a microfluidic application that the printed part may result in retention of liquid during and after the application of flow across the channel. For the same purpose water retention analysis in the microchannel was studied by weight analysis. Once the pressure reading are taken by varying the flowrate through the syringe pump, the inlet port of the microchannel was subjected to the application of compressed air so to remove the access water from the microchannel. After removing the excess water the weight of each microchannel was noted down before and after water flow through the microchannel and the difference (weight variation) between them was calculated. After the experiment, a jet of compressed air was passed through the microchannel to exclude the weight of the stagnant water inside the channel. The weight analysis for each of the design can be observed in the graphs as shown in Figure 10. In each design, the trend shows a linear increase in the weight variation with an increase in the flow rate at all the diameter sizes. Minimum weight variation has been found in the linear microchannel whereas, the maximum variation can be observed in the spiral design. Average weight variation is decreasing with an increase in the diameter size.

**Figure 11:**
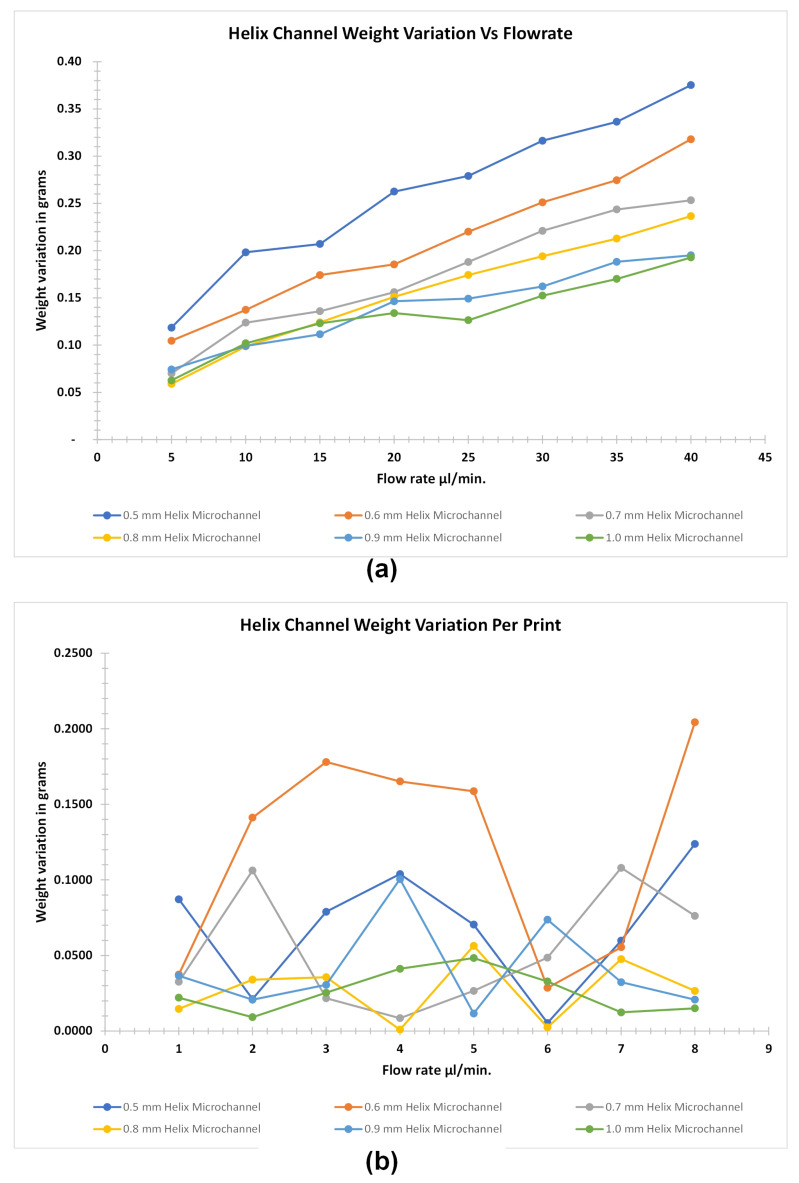
Weight analysis of helical microchannel. (**a**) Water retention with respect to flow rate of diameter size of 500 μm to 1 mm microchannel with an increment of 100 μm (**b**) Weight variation per printing of FDM parts for helical microchannel from 500 μm to 1 mm microholes.

Weight analysis of the helical microchannel is done separately as the size of the microchannels were much bigger than the microchannel printed with different profiles in the preceding section. This type of the microchannel changes its profile in the Z-plane during the FDM printing if we consider the movement of the nozzle for a specific layer in XY plane. The result of the weight analysis of the helical microchannel shows that it is more susceptible to retain water when compared to the other profile. This is due to the application of more pressure to generate the flow. Also, there are more inter layer voids which enhances the seepage of water between the voids. Figure 11 shows the water retention in the helical microchannel profile at various flowrates and the variation of weight of the similar size microchannel for consecutive FDM printed parts. The retention of water is proportional to the applied flowrate since the weight of the parts increases with the increase of flowrate. However, above 900 μm microchannel sizes in out-of-plane geometry, the retention follows the similar profile when compared to smaller microchannel sizes of in-plane geometry.This shows that the constriction in the microchannel for complex out-of-plane geometrical profile above 900 μm in diameter is low as the slicing software was able to map the printed layers with good accuracy when the ratio of layer thickness to the microchannel diameter is 1:10 (in our case we used layer thickness of 100 μm for all the microfluidic channel) or greater for complex out-of-plane geometry. This is due to the constant backlash speed of the printing nozzle with respect to the rapid profile changes in the out-of-plane geometry of the microfluidic channel. And such changes cannot be mapped exactly by varying the backlash speed in realtime for the two low-cost FDM printer through their respective slicing software used in this study. Hence, we can establish that the complex out-of-plane geometry not only generates more pressure for similar applied flowrate when compared to in-plane geometry microfluidic channel but it is also difficult for the slicing software to exactly replicate the 3D modeled microfluidic profile. It is proposed that the further improvement of the microchannel constriction can be reduced by decreasing the layer thickness however, such can lead to drastic increase in the printing time and eventually increases the cost of fabricated part rendering the FDM printing less feasible for fabricating out-of-plane microchannel profiles having diameter less than 500 μm in size.

**Figure 12:**
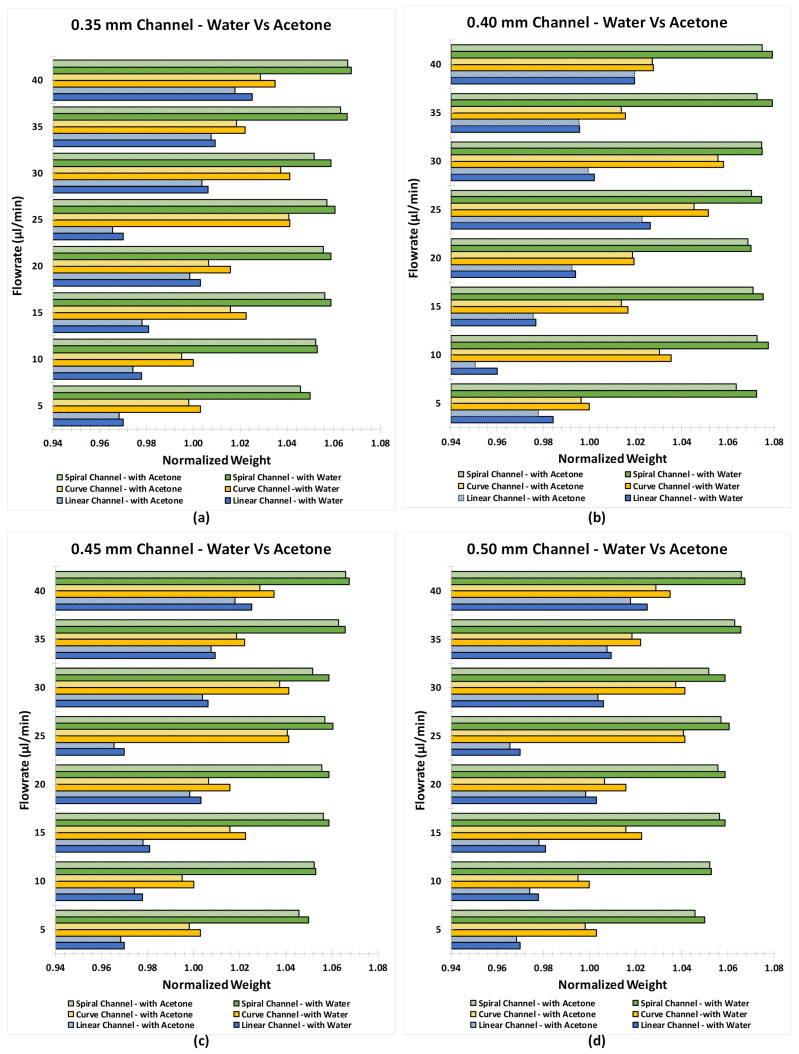
Post-treatment normalized weight analysis of linear, curved and spiral microchannel with diameter sizes of (**a**) 0.35 mm, (**b**) 0.4 mm, (**c**) 0.45 mm, and (**d**) 0.5 mm.

To observe the effect of acetone cleaning/treatment on the inner surface of the microchannel, pre and post-treatment weight analysis was carried out. Also, the post-treatment leakage through the microchannels was studied. The pre and post-treatment weight of the microchannels were normalised and then plotted as shown in Figure 12. It has been observed that the post-treatment weight of the channels is lesser than the pre-treatment weight. It has also been found that the weight variation is less when the diameter is small and the flow rate is low, and the variation is more when the diameter is large and the flow rate is high. This means that the cleaning of FDM channel with acetone involves the chemical reaction between additives in PLA and acetone which results in the weight loss of the printed FDM parts suggesting that the PLA filament from Tiertime is not a virgin or pure PLA material. As the flowrate of the acetone is increased the loss in the weight is greater than the printed channel subjected to lower flowrates.

When water was passed through the acetone cleaned/treated microchannels, the leakage was detected from the microchannels which were previously observed to be good carriers of water at every flow rate (0.35, 0.4, 0.45, and 0.5 mm). This means that the chemical reaction between the additives in PLA and acetone was unable to prevent the leakage of water from the body of the microchannel.

## 4. Conclusions

In this paper, we demonstrated the possibility to fabricate the internal features of the microfluidic device using FDM printer by optimizing the printer’s parameters. We successfully printed the linear, curved and spiral microchannels with the diameter less than 0.5 mm where the path of the microchannel was along the print layer. The work also demonstrates the limitation of low budget FDM printer fabrication of microchannels where the path of the microchannel follows perpendicular or spline geometry with respect to the print layer. Print resolutions of two different FDM printers were compared and assessed for the suitability of the microchannel printing. One of the selected FDM printer can be used with the widely available open-source slicer and other had the proprietary slicer. The results showed that the low budget FDM printer can print the inline or parallel geometry with better print accuracy and can be used for such type of microchannel channels. However, when the geometry is complex and is not along the print layers the size of the microchannels needs to be increased for milifluidic application. Moreover, the experimental results illustrated the correlation of internal feature (microchannel diameter) with the flow rate and also reflects the maximum pressure of the fluid to which microfluidic device can withstand. Also, it has been observed that water retention in both the designs increased with increasing the diameter size. However, it has been found that the more water retention occurred in the spiral microchannels when compared with the linear and curved microchannels. Furthermore, inner surface cleaning/treatment of the microchannel with acetone was also carried out at different flow rates. After the acetone treatment, it was expected that the internal voids in the channels would be sealed and better results would be observed. However, the results of the porosity test after acetone treatment didn’t support this idea. Thin-walled microchannel was assumed to be the reason for failure in this case. Weight variation in the microchannels was discussed after being treated by acetone. The accuracy of the printer was found to be excellent with just 0.6% variation in both designs.

## Figures and Tables

**Table 1:** Comparison of prominent additive manufacturing and other microfluidic fabrication techniques.

Fabrication Technique for Microfluidics	Process	Typical Microfluidic Resolution (μm)	Advantages	Disadvantages
Stereolithography	Point by Point laser scanning and curing of photocurable resin	10–200 [38,39,40]	High resolution, can produce smoother and large parts with the capability of fabricating internal channels of microfluidics	High capital cost of equipment, photocurable raw material is often expensive and unsuitable for bio applications, printing parameters are intricate, and the equipment requires calibration for consistent print results
FDM	Layer-by-layer deposition of thermoplastic polymer extruded by applying heat and extrusion force to form 3D-printed features and microchannels	400–1000 [41,42]	Low cost of equipment, printing material is biocompatible and low cost. Can produce highly complex internal microchannels with moderate sub millimetre resolution	Limited printing resolution and material often not suitable for high temperature applications
Selective Laser Sintering	Point by Point laser induced sintering of ceramic or metal particles to produce 3D parts	100-600 [43]	Strength of parts is good, can produce parts with higher resolution and fabricated parts can be used in high temperature applications	High capital cost of equipment. Due to sintering the surface is rough, cleaning of internal microchannels is difficult
Soft Lithography	Stamping and moulding of elastomeric materials such as PDMS through contact printing, stamping or master mould imprinting [44].	0.05–0.5 [45]	Produces high resolution microchannels, fabricated devices are biocompatible [40]	Permeable for certain liquids and microchannels can swell due to reaction with certain chemicals. Multistep process, stripping of mould requires precision and expertise. Fabrication of complex 3D microchannel is difficult [46]
Inkjet Printing	Drop-on-demand (DoD) piezo-inkjet printing of ink which can be used to form open channel microfluidic, to form microfluidic path for positive or negative photoresist to use for etching or to form layer by layer adhesive structure for microfluidic applications	42–300 [47]	Low cost, high speed, configurable and multimaterial parts can be manufactured [48].	Strength of fabricated devices is low for enclosed channels and fabrication of internal microchannels requires further processing or processes
Xurography	Shearing of material through a sharp knife controlled through a computer-controlled software instructions [17].	15–250 [18,19]	Low cost of equipment, high speed and large area fabrication.	Complex 3D microchannel is extremely difficult to produce.

**Table 2:** Printer specifications and selected parameters.

Specifications	Tiertime UP 02	Prusa i3 MK3S
Make	Tiertime	Prusa Research
Software	UP Studio	Prusa Slicer and Pronterface
Machine Dimensions	245 × 350 × 260 mm	550 × 400 × 500 mm
Build Volume	140 × 140 × 135 mm	250 × 210 × 210 mm
Material Used	PLA (1.7 mm)	PLA (1.7 mm)
Nozzle diameter	0.4 mm	0.4 mm
Support Structure	No Support	No Support
Layer Thickness	100 μm	100 μm
In-Fill	100%	100%
Price	799 USD [54]	749 USD [55]

**Table 3:** Surface roughness profile parameters.

Parameter	Straight	Curved	Spiral	Helical
(All Values Are in μm)	Channel	Channel	Channel	Channel
Roughness Average, $R_{a}$	0.598	1.817	2.170	2.907
Average Maximum Height of the Profile, $R_{z}$	4.013	11.334	13.343	16.396
Maximum Height of the Profile, $R_{t}$	4.888	13.146	16.740	21.784
RMS Roughness, $R_{q}$	0.794	2.241	2.713	3.570
Maximum Profile Peak Height, $R_{p}$	1.147	6.071	5.906	6.575
Kurtosis, $R_{ku}$	4.787	2.712	3.049	3.876
Skewness, $R_{sk}$	1.137	0.150	0.524	0.750

**Table 4:** Pressure developed in microchannels vs. flow rate. The cells highlighted in red indicate the pressure at which the microchannels started to leak.

Microchannel Design	Flow Rate (μL/min)	Pressure (Pa) Microchannel Size (mm)			
		0.35	0.4	0.45	0.5
Linear	5	862	517	0	0
	10	1724	517	517	345
	15	2068	1379	862	517
	20	3275	2241	1034	1034
	25	3620	3103	1724	1551
	30	4482	3964	2758	2413
	35	5171	4482	3447	3275
	40	6033	5516	4137	3964
Curve	5	1034	552	207	207
	10	2068	621	965	827
	15	3447	2068	1517	1724
	20	4275	3006	2068	2206
	25	5171	3,861	2827	2551
	30	5447	4068	3103	3309
	35	6205	4826	4068	4551
	40	6688	5998	4344	4826
Spiral	5	1517	676	138	345
	10	2620	827	1379	1241
	15	4068	2965	2413	1999
	20	4482	3861	2482	2689
	25	5309	4619	3275	3103
	30	5998	5171	3723	3654
	35	7757	6067	4551	4688
	40	8618	6550	4826	5516

## Abbreviations

The following abbreviations are used in this manuscript:

3DP	Three dimensional printing
FDM	Fused deposition modeling
SLS	Selective laser sintering
SL	Stereolithography
PDMS	Polydimethylsiloxane
PLA	polylactic acid
TEB	total error band
FSS	full-scale span
UP02	Tiertime UP 02
Prusa	Original Prusa i3 MK3S
